# Child Marriage and Sexual Autonomy among Women in Sub-Saharan Africa: Evidence from 31 Demographic and Health Surveys

**DOI:** 10.3390/ijerph18073754

**Published:** 2021-04-03

**Authors:** Eugene Budu, Bright Opoku Ahinkorah, Abdul-Aziz Seidu, John Elvis Hagan, Wonder Agbemavi, James Boadu Frimpong, Collins Adu, Kwamena Sekyi Dickson, Sanni Yaya

**Affiliations:** 1 Department of Population and Health, University of Cape Coast, Cape Coast PMB TF0494, Ghana; budueugene@gmail.com (E.B.); abdul-aziz.seidu@stu.ucc.edu.gh (A.-A.S.); wonder.agbemavi@stu.ucc.edu.gh (W.A.); kwamena-sekyi.dickson@stu.ucc.edu.gh (K.S.D.); 2 School of Public Health, Faculty of Health, University of Technology Sydney, Sydney, NSW 2007, Australia; brightahinkorah@gmail.com; 3 College of Public Health, Medical and Veterinary Services, James Cook University, Townsville, QLD 4811, Australia; 4 Department of Health, Physical Education, and Recreation, University of Cape Coast, Cape Coast PMB TF0494, Ghana; frimpongboadujames@gmail.com; 5 Neurocognition and Action-Biomechanics-Research Group, Faculty of Psychology and Sport Sciences, Bielefeld University, Postfach 10 01 31, 33501 Bielefeld, Germany; 6 Department of Health Promotion, Education and Disability Studies, Kwame Nkrumah University of Science and Technology, Kumasi PMB AK, Ghana; collinsadu80@yahoo.com; 7 School of International Development and Global Studies, University of Ottawa, Ottawa, ON K1N 6N5, Canada; 8 The George Institute for Global Health, Imperial College London, London W12 0BZ, UK; sanni.yaya@uOttawa.ca

**Keywords:** child marriage, intimate partner violence, public health, sexual autonomy, SSA

## Abstract

Child marriage has a variety of undesirable consequences at the peril of women’s health and autonomy. In this study, we examined the association between child marriage and sexual autonomy among women in sub-Saharan Africa. We utilised data from the most recent Demographic and Health Surveys conducted in 31 countries in sub-Saharan Africa between 2010 and 2019. A total of 218,578 women aged 20–49 were included in this study. Multivariable binary logistic regression models were used to show the association between child marriage and sexual autonomy. Crude odds ratio (cOR) and adjusted odds ratio (aOR) were used in presenting the results. The prevalence of child marriage and sexual autonomy was 44.51% and 83.35%, respectively. Compared to women who married at 18 years or above, those who married at less than 18 were less likely to have sexual autonomy, and this persisted after controlling for important covariates. In terms of the country-specific results, women who experienced child marriage were less likely to have sexual autonomy in Burundi, Congo DR, Nigeria, and Niger. With the covariates, lower odds of sexual autonomy were found among women with no formal education, those whose partners had no formal education, those who were not exposed to media, and non-working women. Child marriage was found to be associated with sexual autonomy. There is a need to strengthen policies and programmes such as compulsory basic education, poverty alleviation, and an increase in access to media that aim at reducing child marriage. These interventions will help to improve sexual autonomy among women, especially in this 21st century where individuals and organisations incessantly advocate for gender equality.

## 1. Introduction

The United Nations International Children’s Emergency Fund (UNICEF) has emphatically stated that child marriage is a violation of human rights since it deprives individuals of many opportunities such as the right to health, safety, and education [1]. It also has adverse effects on the child, especially girls, and future children, leading to an intergenerational cycle of disadvantages [2]. Child marriage is referred to as a marriage occurring when one of the spouses is younger than 18 years or both are less than 18 years at the time of marriage [3]. Child marriage is a key driver of adolescent childbearing. For instance, 90% of adolescent pregnancies in the developing world are married girls [4]. This trend is because early marriage is likely to reduce sexual autonomy [5]. Sexual autonomy is the right to determine when, with whom, and under what circumstances one can engage in sexual activity [6]. Child marriage does not only reduce sexual autonomy but also reduces the child’s general autonomy.

The global number of child brides is now estimated at 650 million, including girls under age 18 who have already married, and adult women who married in childhood [7]. About 12 million girls are married before age 18 every year [1]. It is estimated that more than 120 million girls will marry before their 18th birthday by 2030. Regional rates of child marriage are highest in South Asia (285 million, 44%) and sub-Saharan Africa (SSA) (115 million, 18%), and low in the Middle East and North Africa (35 million, 5%) [7]. While the global reduction in child marriage is to be commended, no region is on track to meet the Sustainable Development Goal (SDG) target of eliminating this harmful practice by 2030, suggesting that this practice remains a major challenge since the lives of many young people may be at risk.

All African countries are faced with the challenge of child marriage. According to a report by Girls, not Brides, the number of girls married as children will double by 2050 and Africa will become the region with the highest prevalence if no pragmatic interventions are taken [8,9]. Available statistics show that Niger has the highest overall prevalence (76%) of child marriage in Africa and the world at large while Algeria has the lowest of 2 per cent in Africa [1]. In SSA, 35% of young women were married before the age of 18 [1] and the global burden of child marriage is shifting from South Asia to SSA [10]. This shift may have multiple implications on the girl child.

Contextually, even though the most common forms of marriage tend to vary per country, historical, and religious antecedents show that Islamic and customary marriages are fairly similar across African nations [11]. Both types of marriages allow for polygamy, which may include child marriage [11]. Apart from the statutory marriage system, there is no prescribed age of marriage regarding customary and Islamic marriages [11].

Literature has shown that child marriages are most common in rural areas, with poverty, cultural traditions, and values based on patriarchal norms cited as fundamental reasons for the practice [12,13,14]. The low level of education of girls, the lower status of girls, the consideration of girls as a financial burden, social customs, and a mixture of these causes result in the imprisonment of girls in marriage without consent [15]. Montazeri et al. [14] also reported that freedom from undesirable and rigid rules of parents, the low authority of girls, and lack of power to make a decision are also some notable consequences of child marriage. A variety of reported undesirable consequences against women’s health and autonomy include girls’ mobility, inadequate families’ commitment to girls’ schooling, inadequate access to health services, lack of consent to sex, and limited opportunities for social engagement or work opportunities outside the household [16,17].

Despite numerous studies [18,19,20] measuring women’s autonomy on household-level decision-making about their health care and large household purchases and the ability to decide to visit family, limited attention has been given to explicit measures such as sexual autonomy compared to women’s general autonomy [21]. In SSA, this neglect could be attributed to the issues surrounding marriage, divorce, and sexual autonomy. Specifically, in many countries in SSA, there are adverse societal reactions levelled against divorce, preventing women from obtaining the personal freedom, liberty, and autonomy they desire, including sexual autonomy [22]. These few studies found that high sexual autonomy lowers the risk of unwanted pregnancy [23], increases the use of modern contraception [24], and also impacts other reproductive health decisions (e.g., when to have sex, next birth, deciding to use a contraceptive) [25]. Of these few studies that exist, there seems to be little information on the connection between child marriage and sexual autonomy among women. The purpose of this study was to examine the association between child marriage and sexual autonomy among women in SSA. We hypothesised that women who married before 18 years are less likely to have sexual autonomy, compared to those who married after 18 years.

## 2. Material and Methods

### 2.1. Study Design

This study utilised data from the Demographic and Health Surveys (DHS) conducted in 31 countries in SSAs between 2010 and 2019. The DHS uses a repeated cross-sectional research design in carrying out nationally representative survey in over 85 low-and middle-income countries globally. It focuses on essential maternal and child health markers such as child marriage and sexual autonomy [26].

### 2.2. Data Collection Procedure

The data collection procedures for the surveys involve the use of a standard questionnaire comparable across countries for gathering data from women aged 15–49 and men aged 15–59 as well as data on their children. The questionnaire is often translated into the major local languages of the countries involved. To ensure the validity of the translated questionnaires, the DHS reports that the translated questionnaires, together with the version in English are pretested in English and the local dialect. After that, the pre-test field staff actively discussed the questionnaires and made suggestions to modify all versions. Following field practice, a debriefing session is held with the pre-test field staff, and modifications to the questionnaires are made based on lessons drawn from the exercise. Details of the sampling methods, procedures, and implementation can be found on the DHS website in each country’s final report [27].

### 2.3. Sampling Procedure and Size

The sampling procedure employed in the surveys involves a two-stage stratified sampling procedure, where countries are grouped into urban and rural areas. The first stage involves the selection of clusters usually called enumeration areas (EAs) and the second stage consists of the selection of a household for the survey. The study by Aliaga and Ruilin [27] provides details of the sampling process. In this study, a total of 218,578 women who had complete information on all the variables of interest were included in the study (Table 1). We relied on the Strengthening the Reporting of Observational Studies in Epidemiology (STROBE) statement in writing the manuscript [28]. The dataset is freely available for download at https://dhsprogram.com/data/available-datasets.cfm (accessed on 17 February 2021)

### 2.4. Definition of Variables

#### 2.4.1. Outcome Variable

The outcome was sexual autonomy, which was a composite variable derived from “respondent can refuse sex,” “respondent can ask partner to use condom,” and “wife is justified in asking the husband to use condom.” The response categories of these variables were: “Yes” and “No”. The “Yes” responses were coded “1” and the “No” responses were coded “0”. An index was created with all the “Yes” and “No” answers with scores stretching from 0 to 3. The score 0 was labelled as “No” and 1 to 3 was labelled as “Yes”, where “No” represents females who did not have sexual autonomy and “Yes” if females had sexual autonomy [29].

#### 2.4.2. Independent Variables

The study used child marriage as the key independent variable. This variable was derived from the question, “at what age did [NAME] first enter marriage or cohabitation. The response to this question was in single years. For this study, the response was categorised into “married less than 18 years” = 1, where respondents stated they first entered marriage or cohabitation before the age of 18 years, and “married 18 years or more” = 2 for respondents who married or cohabited after 17 years.

### 2.5. Statistical Analyses

Data were analysed with Stata version 16.0 (StataCorp LLC, College Station, TX, USA). The analysis was done in four steps. The first step was a graphical representation of the prevalence of child marriage and the prevalence of sexual autonomy in SSA. The second step was a bivariate analysis that calculated the proportion of sexual autonomy across the explanatory variables with their *p* values which were derived from a chi-square of fitness. All the variables that showed statistical significance from the chi-square test were moved to the third step of the analysis. In the third step of the analysis, two hierarchical logistic regression models were built. The model I looked at a bivariate analysis between the independent variables and sexual autonomy. Model II controlled for the effect of all the independent variables in amultivariable logistic regression. In the fourth and final step of the analysis, a logistic was fitted to see the effect of child marriage on sexual autonomy, disaggregated by country. With this, two models were fitted where Model I was the crude odds ratio (cOR) and Model II was the adjusted odds ratio (aOR). The choice of the reference categories for the regression models was influenced by the sample sizes, with categories with the highest sample sizes chosen as reference categories. All frequency distributions were weighted, while the survey command (svy) in Stata was used to adjust for the complex sampling structure of the data in the regression analyses.

### 2.6. Ethical Approval

The DHS reports that ethical clearances were obtained from the Ethics Committee of ORC Macro Inc. as well as Ethics Boards of partner organisations of the various countries such as the Ministries of Health. The DHS follows the standards for ensuring the protection of respondents’ privacy. Inner City Fund International ensures that the survey complies with the United States Department of Health and Human Services’ regulations for the respect of human subjects. Since this was a secondary analysis, no further ethical approval was required because the datasets are available for download in the public domain. Further information about the DHS data usage and ethical standards is available at http://goo.gl/ny8T6X (accessed on 17 February 2021)

## 3. Results

### 3.1. Prevalence of Child Marriage and Sexual Autonomy in Sub-Saharan Africa

**Figure 1:**
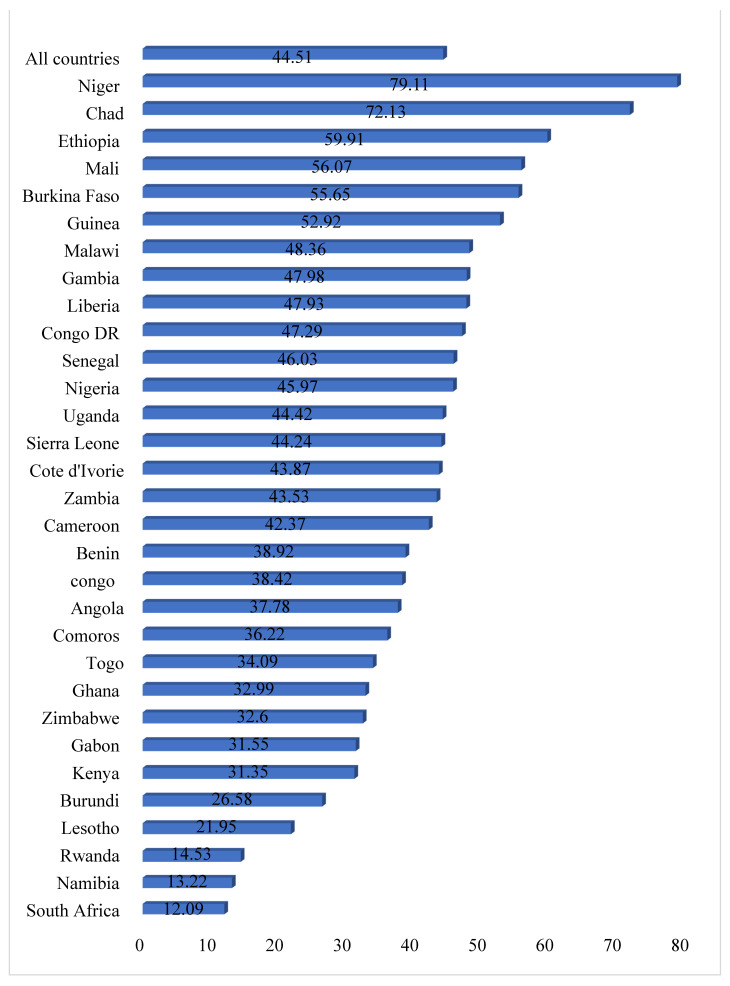
Bar chart showing the prevalence of child marriage in sub-Saharan Africa.

**Figure 2:**
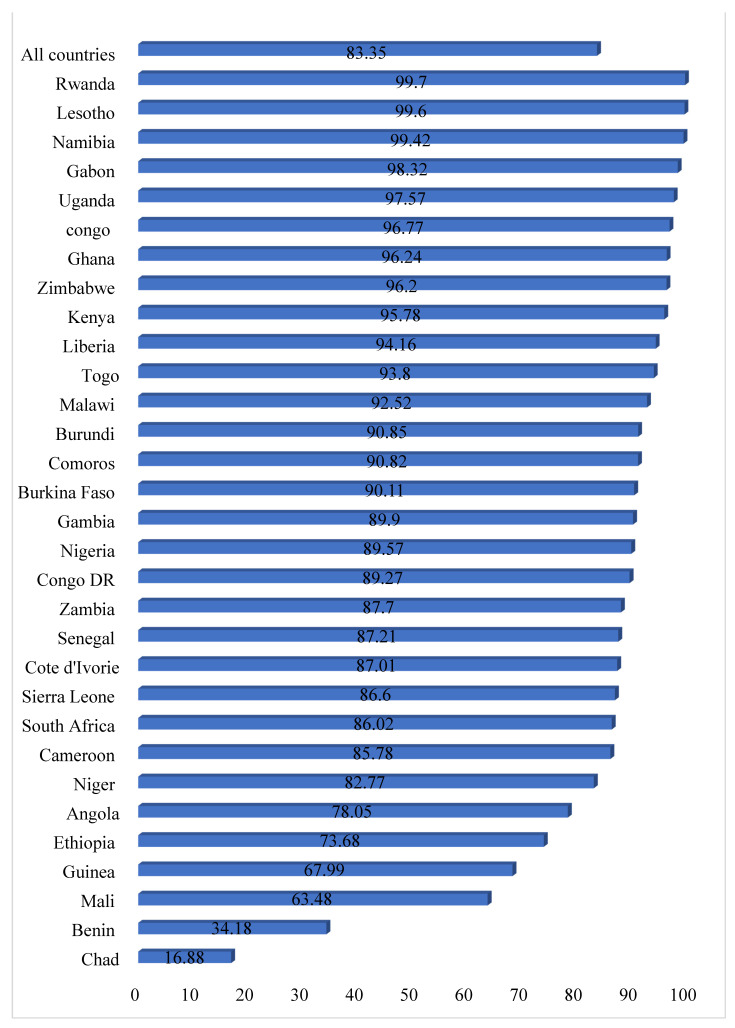
Bar chart showing the proportion of women who have sexual autonomy in sub-Saharan Africa.

Figure 1 presents results on the prevalence of child marriage among women in sub-Saharan African countries. On average, the prevalence of child marriage was 44.51%. The highest prevalence was among women in Niger (79.11%) while the lowest prevalence was in South Africa (12.09%). In terms of the proportion of women who had sexual autonomy, an average prevalence of 83.35% was recorded in all the countries considered in this study, with the highest and lowest prevalence in Rwanda (99.70%) and Chad (16.88), respectively (Figure 2).

### 3.2. Distribution of Sexual Autonomy across Age at First Marriage and Other Socio-Demographic Characteristics of Women

We found a significant association between age at first marriage and sexual autonomy with women who experienced child marriage having a lower prevalence of sexual autonomy (79.2%), compared to those who married as adults (86.7%). In terms of the covariates, there were significant variations in sexual autonomy across the socio-demographic characteristics of the women (mother’s age, educational level, employment status, mass media exposure, partner’s educational level, type of place of residence, wealth quintile, and sex of the household head) (Table 2).

### 3.3. Association between Age at First Marriage and Sexual Autonomy in Sub-Saharan Africa

Table 3 shows results on the association between age at first marriage and sexual autonomy in sub-Saharan Africa. We found that compared to women who married at 18 years or above, those who married at less than 18 were less likely to have sexual autonomy (cOR = 0.58, CI: 0.56–0.60) and this persisted after controlling for important covariates (aOR = 0.86, CI: 0.83–0.89). With the covariates, higher odds of sexual autonomy were found among women aged 35–39, compared to those aged 25–29 (aOR = 1.14, CI: 1.08–1.19); those with secondary/higher education compared to those with primary education (aOR = 1.39, CI: 1.30–1.48) and women in female-headed households compared to those in male-headed households (aOR = 1.13, CI: 1.07–1.19) (see Model II of Table 3). Lower odds of sexual autonomy were found among women with no formal education (aOR = 0.50, CI: 0.47–0.53), those whose partners had no formal education (aOR = 0.56, CI: 0.53–0.60), and those who were not exposed to media (aOR = 0.53, CI: 0.50–0.55), and non-working women (aOR = 0.62, CI: 0.59–0.65) (see Model II of Table 3). 

In terms of the country-specific results, women who experienced child marriage were less likely to have sexual autonomy in Burundi (aOR = 0.78, CI: 0.67–0.92), Congo DR (aOR = 0.86, CI: 0.76–0.97), Nigeria (aOR = 0.85, CI: 0.76–0.95), and Niger (aOR = 0.75, CI: 0.64–0.89) (see Model II of Table 4).

## 4. Discussion

Child marriage is a violation of human rights since it deprives the individuals involved of many opportunities such as the right to health, safety, and education [1]. The study examined the associations between child marriage and sexual autonomy among women using data from DHS conducted in 31 sub-Saharan African countries. This study found that the prevalence of child marriage was 44.5% and that of sexual autonomy was 83.4%. Age at first marriage, mother’s age, educational level, employment status, mass media exposure, partner’s occupational status, type of place of residence, wealth quintile, and sex of head of household were found as factors associated with sexual autonomy.

The prevalence of child marriage (44.5%) recorded in this study was lower than what was recorded in other studies in SSA (54.0%) [17] and Bangladesh (78.2%) [30]. However, substantial variations were detected as Niger had the highest (79.1%) prevalence with South Africa recording the lowest (12.1%) prevalence. A possible reason for the variation in the prevalence among the countries could be as a result of the disparity in socio-cultural practices and beliefs regarding child marriage. For instance, it could be that the practice of child marriage is still being upheld in Niger, which is reflected in its high prevalence rate [17]. Other reasons that may account for the high prevalence of child marriage in sub-Saharan Africa could be the dominance of customary and Islamic marriages, which usually have no age limit for marriage in the sub-region [11]. This suggests that more efforts are needed in the fight against child marriage in the sub-region.

This study also found 83.4% prevalence of sexual autonomy among women in the selected countries in SSA. There were regional disparities in the prevalence of sexual autonomy among women in SSA. While Rwanda had the highest (99.7%) sexual autonomy, Chad recorded the least (16.9%) sexual autonomy among women. A possible explanation for this finding could be that compared to Chad, women in Rwanda have been empowered to make decisions regarding their sexual life [31]. Another possible reason for the low prevalence of sexual autonomy in Chad could be linked to the socio-cultural norms around divorce in several countries in SSA, including Chad. Since divorce is infringed upon, women in the country are likely to lose their autonomy to negotiate for safer sex in order not to get divorced [22]. The finding suggests that countries in SSA are still deprived of the necessities that lead to improved sexual autonomy among women. Therefore, policies and interventions that empower women to make critical and affirmative decisions regarding their sexuality in such countries are in the right direction in this 21st century where gender equality is mostly advocated.

Similar to observations from other previous studies [5,32,33,34,35] we found that women who married at less than age 18 were less likely to have sexual autonomy compared to those who married at 18 years or above. An acceptable explanation that could be given to this finding is that women who marry early may be from traditional family settings and are deprived of taking important decisions about their own life such as marriage, which are mostly arranged for them [5,35,36]. Another reason could be that women who marry early may lack the ability to resist any actions from their spouse such as the use of contraceptives and child spacing when it comes to sexual engagements [21]. The finding indicates that health interventions that improve women’s sexual autonomy have neglected those that marry before age 18, hence, they should be considered in health promotion interventions.

This study also found that women who experienced child marriage were less likely to have sexual autonomy in Niger, Burundi, Nigeria, and Congo DR. A possible reason for this finding could be associated with the fact that married women who are below the age of 18 may lack the ability to speak out for their sexual rights since their male partners are the ones that take most of the decisions in the house, including decisions of sex [20,33]. This suggests that continuous public health education is needed to inform countries in SSA about the health consequences of child marriage on women’s sexual autonomy.

Corroborating the observation of previous studies [20,32,33], we found lower odds of sexual autonomy among women with no formal education. A possible reason for this finding could be that since schools provide people with the opportunity to be informed about sexual issues through health education and promotion, women who have no formal education may lack certain vital information that could empower them to be sexually autonomous [20]. For example, women who have no formal education may not be adequately informed about contraceptives such as female condoms and how they are used as well as the negative health implications of untimely birth. This finding suggests that health promotion interventions that are aimed at improving women’s sexual autonomy should target uneducated women if further progress is to be made.

Akin to the finding of a previous study [33], this study found lower odds of sexual autonomy among women whose partners had no formal education. An acceptable explanation for this finding could be that since men with no formal education may be less informed about sexual issues, they may prevent their wives from making decisions that could affect their health as well [33]. For instance, a man who has no knowledge of condom use may likely decline the wife’s proposal for using condoms during sex. The finding suggests that to achieve success in the improvement in sexual autonomy among women in SSA, men with no formal education should also be targeted in health promotion interventions that help to improve women’s autonomy regarding their sexuality.

We found lower odds of sexual autonomy among women who were not exposed to mass media. A possible reason for this finding could be that since mass media serve as an avenue for rolling out health education and promotion programmes for varied populations at the same time, women who have no access to the media may not be privy to some information that could play significant roles in making decisions about their sexual life. The finding suggests that other means such as community outreach should be used alongside the mass media to reach out to those women who may not have access to mass media because of other reasons, as this helps raise the sexual autonomy of such women.

Similar to previous studies [20,33,37], lower odds of sexual autonomy were found among women who were not working compared to those who were working. A possible reason for this finding could be that women’s ability to make decisions about their sexual life enhances when they are employed [33]. This is so because they can afford any health service when they are employed as compared to those that are not working. This finding indicates that to improve sexual autonomy among women in SSA, they should be provided with jobs. Hence, health promotion organisations and interventions that help in increasing women’s sexual autonomy should also consider creating jobs for women as this will help increase their sexual autonomy.

### 4.1. Limitations

The study was limited to the variables available in the DHS datasets for the analysis. The cross-sectional nature of the data restricts causal connections between child marriage and sexual autonomy. The survey was also based on retrospective self-reports of sampled women, which like in many surveys, such data may be prone to social desirability concerns (e.g., recall bias, over and/or under-reporting). Context-specific variations may mean that women with diverse social or cultural backgrounds may report experiencing child marriage and many forms of behaviour associated with sexual autonomy differently, which might have influenced our current results. Despite these limitations, this study is one of the few that provides empirical linkages between child marriage and sexual autonomy among women in selected sub-Saharan African countries that previous research has ignored. Additionally, the current age of women sampled might explain the high odds of sexual autonomy recorded in the current study.

### 4.2. Practical Implications

Current findings underscore the significance of child marriage as a key indicator associated with sexual autonomy among women in SSA. Women married early are more likely to come from socially disadvantaged or vulnerable populations such as those with no or lesser formal education, those not exposed to media, and not working. Women with these inequalities and in early marriages might be at increased risk for having partners or husbands who are older, abusive, and are more likely to have restrictions on their mobility and decision-making, including their reproductive health decisions (e.g., consent for sex). Given that SDG4 (i.e., education quality), Target 4.5 advocates for the “elimination of gender disparities in education and ensure equal access to all levels of education and vocational training for the vulnerable” and SDG5 (i.e., gender equality), Target 5.3 indicates the “elimination of all harmful practices, such as child, early and forced marriage...” [38], policies and interventions aimed to raise the age of marriage and providing incentives for girls to stay in school are required. Therefore, the elimination or even reduction in child marriage can have a population-specific impact on women’s health and well-being if a greater commitment to the educational development that guarantees retention, continuous support, and value for these disadvantaged women is given priority in SSA. Additional studies are required to investigate the association between women’s decision-making capacity as a mediator between child marriage and sexual autonomy among women in SSA. Using qualitative and longitudinal designs to further explore these linkages could help in the design of appropriate programmes to promote women’s health.

## 5. Conclusions

The findings of this study indicate that the prevalence of child marriage and sexual autonomy among women in selected countries in SSA were 44.5% and 83.4%, respectively. Child marriage was found to be associated with sexual autonomy. There is the need to strengthen policies and programmes such as compulsory basic education, poverty alleviation, and an increase in access to media that aim at reducing child marriage. This will help to help improve sexual autonomy among women, especially in this 21st century where individuals and organisations incessantly advocate for gender equality.

## Figures and Tables

**Table 1:** Description of the Study Sample.

Countries	Survey Year	Weighted N	Weighted %
Angola	2015–16	5788	2.65
Burkina Faso	2010	12,015	5.50
Benin	2018–19	9572	4.38
Burundi	2016–17	9266	4.38
Congo DR	2013–14	9723	4.45
Congo	2011–12	5347	2.45
Côte d’Ivoire	2011–12	4997	2.29
Cameroon	2018	6874	3.14
Ethiopia	2016	8320	3.81
Gabon	2012	3590	1.64
Ghana	2014	4816	2.40
Gambia	2013	5252	2.40
Guinea	2018	5280	2.42
Kenya	2014	7762	3.55
Comoros	2012	2254	1.03
Liberia	2013	4781	2.19
Lesotho	2014	1368	0.63
Mali	2018	6813	3.12
Malawi	2015–16	14,322	6.55
Nigeria	2018	19.293	8.83
Niger	2012	7130	3.26
Namibia	2013	2304	1.05
Rwanda	2014–15	6698	3.06
Sierra Leone	2019	8381	3.83
Senegal	2010–11	7908	3.62
Chad	2014–15	9109	4.17
Togo	2013–14	5408	2.47
Uganda	2016	9673	4.42
South Africa	2016	2265	1.04
Zambia	2018–19	6806	3.11
Zimbabwe	2015	5481	2.51
Total		218,578	100

**Table 2:** Distribution of sexual autonomy across age at first marriage and other socio-demographic characteristics of women.

Variables	Weighted N	Weighted %	Sexual Autonomy	*p* Value
**Age at First Marriage**				<0.0001
Less than 18 years	97,287	44.5	79.2	
18 years and more	121,291	55.5	86.7	
**Mother’s Age**				<0.001
20–24	39,675	18.2	83.0	
25–29	50,806	23.24	83.3	
30–34	44,274	20.26	83.9	
35–39	37,336	17.08	84.0	
40–44	26,124	12.0	83.5	
45–49	20,363	9.3	81.4	
**Educational Level**				<0.0001
No education	89,929	41.1	72.7	
Primary	65,178	29.8	88.4	
Secondary/higher	63,471	29.0	93.3	
**Employment Status**				<0.0001
Not working	55,761	25.5	77.5	
Working	162,817	74.5	85.4	
**Mass Media Exposure**				<0.0001
No	75,782	35.1	74.6	
Yes	141,796	64.9	88.1	
**Partner’s Educational Level**				<0.0001
No education	75,791	34.7	72.1	
Primary education	57,357	26.2	86.7	
Secondary/higher education	85,429	39.1	92.1	
**Partner’s Occupational Status**				<0.0001
Not working	7842	3.6	79.6	
Working	210,736	96.4	83.5	
**Type of Place of Residence**				<0.0001
Urban	77,470	35.4	88.9	
Rural	141,108	64.6	80.3	
**Wealth Quintile**				<0.0001
Poorest	40,315	18.4	77.7	
Poorer	43,168	19.8	80.4	
Middle	43,511	19.9	82.5	
Richer	45,222	20.7	85.4	
Richest	46,362	21.2	89.8	
**Sex of Head of Household**				<0.0001
Male	185,236	84.7	82.8	
Female	33,342	15.3	86.4	

**Table 3:** Binary logistic regression results on the predictors of sexual autonomy among women in sub-Saharan Africa.

Variable	Model I cOR (95% CI)	Model II aOR (95% CI)
**Age at First Marriage**		
Less than 18 years	0.58 *** (0.56–0.60)	0.86 *** (0.83–0.89)
18 years and more	Ref	Ref
**Mother’s Age**		
20–24	0.98 (0.94–1.03)	0.98 (0.93–1.02)
25–29	Ref	Ref
30–34	1.05 (1.00–1.09)	1.07 ** (1.02–1.12)
35–39	1.06 * (1.01–1.11)	1.14 *** (1.08–1.19)
40–44	1.02 (0.97–1.07)	1.13 *** (1.07–1.20)
45–49	0.89 *** (0.85–0.94)	1.05 (0.99-1.11)
**Educational Level**		
No education	0.34 *** (0.32-0.36)	0.50 *** (0.47–0.53)
Primary	Ref	Ref
Secondary/higher	1.72 *** (1.61–1.84)	1.39 *** (1.30–1.48)
**Employment Status**		
Not working	0.55 *** (0.53–0.58)	0.62 *** (0.59–0.65)
Working	Ref	Ref
**Exposure to Mass Media**		
No	0.39 *** (0.37–0.41)	0.53 *** (0.50–0.55)
Yes	Ref	Ref
**Partner’s Educational Level**		
No education	0.25 *** (0.24–0.27)	0.56 *** (0.53–0.60)
Primary education	0.68 *** (0.65–0.72)	0.97 (0.92–1.02)
Secondary/higher education	Ref	Ref
**Partner’s Occupational Status**		
Not working	0.81 *** (0.74–0.89)	0.95 (0.87–1.04)
Working	Ref	Ref
**Type of Place of Residence**		
Urban	1.77 *** (1.63–1.92)	0.98 (0.89–1.07)
Rural	Ref	Ref
**Wealth Quintile**		
Poorest	0.85 *** (0.81–0.90)	1.04 (0.98–1.10)
Poorer	Ref	Ref
Middle	1.14 *** (1.09–1.20)	0.97 (0.92–1.02)
Richer	1.41 *** (1.32–1.51)	0.97 (0.90–1.04)
Richest	2.12 *** (1.93–2.32)	0.93 (0.85–1.03)
**Sex of Head of Household**		
Male	Ref	Ref
Female	1.30 *** (1.23–1.37)	1.13 *** (1.07–1.19)

COR = crude odds ratio; AOR = adjusted odds ratio; CI = confidence interval; Ref = reference category, * *p* < 0.05 ** *p* < 0.01 *** *p* < 0.001.

**Table 4:** Binary logistic regression results on the association between age at marriage and sexual autonomy among women in sub-Saharan Africa by country.

Countries	Model I cOR (95% CI)	Model II aOR (95% CI)
Angola	0.96 (0.85–1.09)	1.14 (0.99–1.30)
Burkina Faso	0.82 *** (0.73–0.93)	0.93 (0.82–1.05)
Benin	0.86 *** (0.79–0.94)	0.93 (0.85–1.02)
Burundi	0.74 *** (0.63–0.86)	0.78 *** (0.67–0.92)
Congo DR	0.77 *** (0.68-.87)	0.86 ** (0.76–0.97)
Congo	0.71 ** (0.54–0.94)	0.77 (0.58–1.05)
Côte d’Ivoire	0.66 *** (0.56–0.78)	0.85 (0.71–1.01)
Cameroon	0.61 *** (0.53–0.70)	0.98 (0.84–1.14)
Ethiopia	0.78 *** (0.70–0.86)	1.05 (0.93–1.7)
Gabon	0.78 (0.46–1.30)	1.25 (0.72–2.18)
Ghana	0.73 ** (0.55–0.98)	1.01 (0.75–1.35)
Gambia	0.78 *** (0.67–0.92)	1.00 (0.84–1.19)
Guinea	0.93 (0.83–1.04)	1.04 (0.92–1.17)
Kenya	0.55 *** (0.47–0.66)	1.03 (0.85–1.26)
Comoros	0.91 (0.68–1.22)	1.11 (0.81–1.52)
Liberia	0.77 ** (0.62–0.97)	0.85 (0.67–1.07)
Lesotho	1.06 (0.22–5.13)	1.59 (0.29–8.79)
Mali	0.90 ** (0.81–0.99)	0.96 (0.86–1.07)
Malawi	0.97 (0.85–1.10)	1.15 (0.99–1.31)
Nigeria	0.41 *** (0.37–0.45)	0.85 *** (0.76–0.95)
Niger	0.62 *** (0.53–0.72)	0.75 *** (0.64–0.89)
Namibia	1.65 (0.38–7.09)	2.76 (0.60–12.81)
Rwanda	0.38 * (0.15–1.00)	0.43 (0.16–1.16)
Sierra Leone	1.07 (0.95–1.22)	1.12 (0.99–1.27)
Senegal	0.78 *** (0.69–0.88)	1.06 (0.93–1.20)
Chad	1.00 (0.88–1.13)	1.10 (0.96–1.36)
Togo	0.60 *** (0.49–0.74)	0.84 (0.68–1.04)
Uganda	0.79 * (0.61–1.01)	1.00 (0.77–1.29)
South Africa	0.72 * (0.51–1.01)	0.76 (0.53–1.08)
Zambia	0.89 (0.77–1.02)	1.10 (0.94–1.27)
Zimbabwe	0.64 *** (0.49–0.84)	0.96 (0.72–1.29)

* *p* < 0.05 ** *p* < 0.01 *** *p* < 0.001. cOR = crude odds ratios; aOR = adjusted odds ratios.

## Data Availability

The dataset is available on the following website: http://goo.gl/ny8T6X (accessed on 12 February 2021).
